# Mental Health and Identity Formation Among Primary School Learners: Peer Perceptions of Children Living with Epilepsy Through a Social-Educational Lens

**DOI:** 10.3390/ijerph22111761

**Published:** 2025-11-20

**Authors:** Thendo Gertie Makhado, Lufuno Makhado

**Affiliations:** 1 Department of Advanced Nursing Sciences, Faculty of Health Sciences, University of Venda, P. Bag X5050, Thohoyandou 0950, South Africa; 2 Department of Public Health, Faculty of Health Sciences, University of Venda, P. Bag X5050, Thohoyandou 0950, South Africa; lufuno.makhado@univen.ac.za

**Keywords:** child mental health, epilepsy stigma, inclusive education, identity formation

## Abstract

Children living with epilepsy face significant stigma in school settings, particularly in rural South Africa, where misconceptions linking epilepsy to witchcraft, evil spirits, or unpredictability lead to fear, shame, and exclusion. This study explored how primary school learners perceive and experience epilepsy-related stigma within the classroom context and examined how such perceptions may influence the mental health and self-conception of peers living with epilepsy. A descriptive–exploratory qualitative design was employed, involving six focus group discussions with 36 learners aged 9–14 years from Grades 4 to 7 in rural schools across Limpopo and Mpumalanga provinces. Data were analyzed thematically using ATLAS.ti software (version 22). Findings revealed that epilepsy was commonly described as a “falling disease,” associated with ancestral spirits or supernatural causes, contributing to peer mockery, isolation, and emotional distress. While some learners expressed empathy and willingness to help, most lacked accurate knowledge about the condition. This study concludes that integrating culturally grounded, age-appropriate epilepsy education into life skills curricula can promote empathy, reduce stigma, and support inclusive identity formation. Embedding such programs in school health policies and teacher training frameworks can strengthen mental health promotion and contribute to equitable health education within the goals of Universal Health Coverage.

## 1. Introduction

Epilepsy remains one of the most prevalent neurological disorders of childhood, affecting approximately 0.5–1% of the global pediatric population, with an estimated 80% of cases occurring in low- and middle-income countries (LMICs) [1,2]. In Sub-Saharan Africa, where access to diagnostic and treatment services is limited, children living with epilepsy face not only the biomedical challenges of the condition but also significant psychosocial burdens [3,4]. Beyond its clinical manifestations, epilepsy profoundly affects children’s psychological and social development due to entrenched stigma and cultural misconceptions [5,6]. These misconceptions often link epilepsy to witchcraft, contagion, or evil spirits, and contribute to discrimination, bullying, and social exclusion within school environments [7,8].

Such stigma is particularly detrimental during late childhood and early adolescence, when children are forming their self-concept and identity [9]. Stigmatizing peer interactions and exclusion can undermine emotional well-being, academic engagement, and self-esteem, creating cycles of isolation and internalized shame [10,11]. The consequences are even more pronounced in rural contexts, where psychosocial support services and inclusive educational frameworks remain underdeveloped [12].

Within the framework of Universal Health Coverage (UHC) and the Sustainable Development Goals (SDG 3.8 and SDG 4), inclusive health education represents a crucial yet underexplored dimension of health equity [13]. Life skills education integrating social, emotional, and health competencies offers a pathway to address epilepsy-related stigma early in life, equipping learners with empathy, accurate knowledge, and practical support strategies [4,14]. However, despite growing evidence of the benefits of early educational interventions for mental health promotion, most rural schools in Sub-Saharan Africa lack curricula that address the psychosocial needs of children with chronic conditions [4,15].

This study, therefore, examines how epilepsy-related stigma influences the mental health and identity formation of primary school learners and explores how life skills education can promote inclusion and resilience. By situating the findings within a social-educational lens, the research contributes to global conversations on equitable education and psychosocial well-being, aligning with the WHO’s call for integrating mental health and disability inclusion within UHC models. This study is conceptually anchored in Goffman’s theory of social stigma, which frames stigma as a socially constructed attribute that discredits individuals and alters their identity from “whole” to “tainted” in the eyes of others [16]. Within school contexts, such labeling processes can lead children living with epilepsy to experience enacted and internalized stigma, reinforcing feelings of exclusion and inferiority. To further understand the developmental implications, this study draws on Erikson’s psychosocial theory of identity formation, particularly the stage of industry versus inferiority, during which children develop a sense of competence and self-worth through social feedback and peer relationships [17]. When stigmatization disrupts these interactions, it can distort self-concept and impede healthy identity consolidation. The integration of these frameworks provides a lens through which to interpret how epilepsy-related stigma intersects with developmental processes of self-definition and belonging in middle childhood.

## 2. Materials and Methods

This section describes the methodological framework used to examine how epilepsy-related stigma influences the mental health and self-concept of primary school learners, and how life skills education can promote inclusion within rural South African school contexts.

### 2.1. Research Design

A descriptive–exploratory qualitative design was adopted to gain an in-depth understanding of children’s experiences and perceptions regarding epilepsy. This design was suitable for exploring complex, context-dependent, and socially constructed psychosocial and cultural phenomena [18,19]. The qualitative approach enabled the researchers to uncover how stigma, social interactions, and identity formation intersect in the daily lives of children living with or exposed to epilepsy-related beliefs.

### 2.2. Study Setting and Participants

The study was conducted in rural public primary schools across the Limpopo and Mpumalanga provinces of South Africa, regions characterized by high levels of health inequality and limited access to psychosocial support services [12]. These communities also reflect broader challenges in the implementation of Universal Health Coverage (UHC), including cultural stigma and resource constraints [20]. Mpumalanga and Limpopo provinces were purposively selected because they encompass diverse cultural and linguistic communities, including Venda, Tsonga, Pedi, Swati, and Ndebele groups. This diversity allowed exploration of how different belief systems, social norms, and local languages influence learners’ understanding and experiences of epilepsy. The two provinces also reflect a range of school contexts, from rural to semi-urban, making them suitable for examining the intersection of culture, education, and stigma in everyday school life.

A purposive sampling strategy was employed to recruit 36 learners (aged 9–14 years) enrolled in Grades 4–7. The participants did not include children diagnosed with epilepsy; rather, the focus was on peers who had observed or interacted with classmates living with the condition. This sampling approach ensured the inclusion of participants who were capable of articulating their experiences, while capturing a range of peer perspectives on epilepsy. The age group was specifically chosen because it represents a developmental stage in which self-concept and peer influence are strongly emerging [11]. Inclusion criteria were school enrollment within the target grades and parental consent.

### 2.3. Approvals and Permissions

Prior to data collection, ethical and administrative approvals were obtained from the University of Venda Research Ethics Committee (SHS/19/PH/37/2101) and from the Provincial Departments of Education in Limpopo and Mpumalanga. Permissions were also granted by local school circuits and principals. Written informed consent was secured from parents or legal guardians, and assent was obtained from all learners.

To safeguard confidentiality, each participant was assigned a unique code, and all personal identifiers were removed during transcription and analysis. All audio recordings and transcripts were stored on a password-protected, encrypted institutional drive accessible only to the researchers and supervisors. Hard-copy consent forms were kept in a locked cabinet in the University’s secure research office. In compliance with institutional data protection protocols, all files will be retained for a period of five years before being securely deleted. The study fully complied with the Declaration of Helsinki and the South African Department of Health’s Ethical Guidelines for Research with Human Participants (2015) [21].

### 2.4. Data Collection

Data were collected through six focus group discussions (FGDs) conducted in Mpumalanga and Limpopo Province Primary schools from August to November 2022. Each group consisted of 5–7 learners and was facilitated in a child-sensitive, interactive format. A semi-structured interview guide explored key domains, including perceptions of epilepsy, stigma, peer interactions, and emotional responses to epilepsy. Discussions were conducted in participants’ preferred local languages (Xitsonga, Sepedi, Tshivenda, and isiSwati). After the interviews, the transcripts were transcribed verbatim and translated into English by language experts. The data collection process was structured around a semi-structured interview guide, which included a series of guiding questions designed to elicit comprehensive responses. Key questions within the guide encompassed topics such as the participants’ understanding of epilepsy and the necessity of incorporating epilepsy into life skills education. Specifically, respondents were asked, “Can you describe what you understand about epilepsy?” and “In your opinion, what are some of the reasons why epilepsy should be included in life skills education?” Probing techniques were employed to encourage participants to provide more in-depth and nuanced information on these critical topics.

Sessions lasted approximately 45–60 min and were audio-recorded with prior consent. Field notes captured contextual observations and non-verbal expressions. All sessions were moderated by trained qualitative researchers experienced in child-centered interviewing. The qualitative protocol ensured emotional safety and encouraged voluntary participation through reassurance, flexibility, and debriefing.

### 2.5. Data Analysis

Transcripts were analyzed thematically using ATLAS.ti 22 software. Two researchers independently coded all transcripts and subsequently met to reconcile and agree on coding decisions. Discrepancies were discussed until consensus was reached. To enhance dependability and confirmability, an audit trail was maintained, and peer debriefing was employed to verify the accuracy of themes. While no statistical inter-coder reliability was computed, analytical agreement was established through iterative reflection and comparison against the raw data. Analysis followed Braun and Clarke’s six-phase approach: (1) familiarization with data, (2) initial coding, (3) generation of themes, (4) review of themes, (5) definition and naming of themes, and (6) reporting [22]. Coding was primarily inductive, allowing themes to emerge from participants’ narratives rather than pre-existing frameworks. Peer debriefing among the research team enhanced analytical reliability.

Themes were triangulated with field notes and interviewer reflections to ensure credibility and trustworthiness [23]. The analysis emphasized both semantic and latent meanings in participants’ accounts to reveal the socio-cultural underpinnings of epilepsy stigma and its impact on mental health.

### 2.6. Ethical Considerations

Strict ethical protocols were observed. Participation was voluntary, and learners were informed of their right to withdraw at any point without penalty. Data confidentiality was maintained through anonymization and secure storage. Sensitive topics, such as fear or exclusion, were addressed supportively, with access to school-based counseling services offered where necessary.

### 2.7. Use of Generative AI

Generative artificial intelligence (GenAI) tools were not used in the design, data collection, or interpretation of this study. GenAI was used solely for language refinement and structural clarity during manuscript preparation, without altering research content or participant-derived findings.

## 3. Results

The thematic analysis of six focus group discussions with 36 primary school learners revealed four major themes capturing children’s perceptions, emotional responses, and social experiences related to epilepsy: (1) Perceptions of Epilepsy as “Falling and Fearful”; (2) Social Exclusion and Internalized Stigma; (3) Emotional Distress and Peer Confusion; and (4) Mental Health Impact and Isolation. Each theme underscores how cultural narratives and peer attitudes shape learners’ understanding of epilepsy and affect the well-being of those living with the condition.

### 3.1. Theme 1: Perceptions of Epilepsy as “Falling and Fearful”

Learners commonly described epilepsy through visual and emotional imagery such as “falling,” “shaking,” or being “attacked by spirits.” Misconceptions were rooted in traditional beliefs linking epilepsy to witchcraft, curses, or ancestral anger. These accounts reflect a limited biomedical understanding of the condition and pervasive community myths. The following are the narratives.

*“Epilepsy is a disease that is scary of falling.”*

(Participant A, Focus Group 4).

*“I know is a disease that involves one falling because of evil spirits.”*.

(Participant E, Focus Group 4).

*“When someone has epilepsy, they fall like they were struck by electricity.”*

(Participant B, Focus Group 1).

Such perceptions created fear and confusion among peers, leading to avoidance rather than support when seizures occurred. These narratives illustrate how deeply ingrained beliefs perpetuate stigma from early childhood [24].

### 3.2. Theme 2: Social Exclusion and Internalized Stigma

Learners often explained that children living with epilepsy are treated differently by their classmates. Many are left out of games, teased, or even avoided because others believe the condition is contagious or frightening. As a result, these children begin to feel ashamed and prefer to stay by themselves. Over time, being excluded and ridiculed makes them lose confidence and start to withdraw from social activities, which leads to feelings of loneliness and isolation.

*“Yes, I once saw someone here at school falling during prayer, and people laughed because they said she had demons.”*

(Participant F, Focus Group 5).

*“My friend has it here at school, but she no longer comes because she is always falling and other learners laugh at her.”*

(Participant D, Focus Group 5).

*“Some people don’t want to be friends with someone with epilepsy because they are scared to get it also.”*

(Participant A, Focus Group 4).

Social rejection led to diminished classroom participation and reinforced negative identity formation. Similar findings have been reported among adolescents in other LMICs, where epilepsy stigma impedes social integration [25].

### 3.3. Theme 3: Emotional Distress and Peer Confusion

Learners described fear, panic, and helplessness when witnessing seizures. While some expressed empathy and a desire to help, most lacked knowledge about safe or appropriate responses. The following narratives are from the learners.

*“I just stand scared because I don’t know what to do.”*

(Participant B, Focus Group 3).

*“I ran away because I was scared.”*

(Participant A, Focus Group 5).

This theme highlights both an emotional gap and an educational gap, suggesting that early health literacy interventions could equip children with coping and first-aid skills [8].

### 3.4. Theme 4: Mental Health Impact and Isolation

Children with epilepsy were perceived as withdrawn, anxious, or absent from school. Peers associated frequent absences and sadness with “fear of falling,” reinforcing exclusion.

*“She no longer comes to school because she is always falling, and other learners laugh at her.”*

(Participant D, Focus Group 5).

*“Other learners laugh at me because they say I am a friend of a falling person.”*

(Participant D, Focus Group 5).

This pattern illustrates how stigma, ridicule, and peer rejection collectively erode the self-esteem and emotional well-being of affected learners, aligning with findings from other rural contexts [26].

## 4. Discussion

The thematic analysis of six focus groups with 36 primary school learners reveals that epilepsy-related stigma profoundly affects children’s mental health, peer relationships, and identity formation. The four interlinked themes—perceptions of epilepsy as “falling and fearful,” social exclusion and internalized stigma, emotional distress and peer confusion, and the mental health effects of isolation—form a self-reinforcing cycle of misunderstanding and exclusion that undermines well-being. Central to this cycle is a pervasive lack of knowledge and a cultural narrative portraying epilepsy as dangerous and shameful, leaving affected learners marginalized.

Perceptions of epilepsy as linked to falling, shaking, or spirit possession reflect cultural mythology rather than biomedical understanding. Similar misconceptions persist across rural African and Asian contexts, where epilepsy is often associated with witchcraft or curses [27,28]. These beliefs evoke fear and avoidance, as seen in statements like “I ran away because I was scared,” illustrating how stigma stems from ignorance rather than malice. This miseducation mirrors what Clifford, Brothers, and Lang [29] term “perceived stigma schemas,” where children interpret health differences as threats to belonging.

Fear-driven avoidance evolves into ridicule and ostracism, leading children with epilepsy to internalize stigma and withdraw socially. Moosa [30] observed similar dynamics in South African schools, where learners described themselves as “falling people.” Such rejection fosters internalized shame, eroding confidence and belonging—core needs in childhood. Consequently, social stigma and self-stigma reinforce each other, deepening isolation.

Peers’ confusion during seizures—“I just stand scared because I don’t know what to do”—reflects both limited health literacy and emotional contagion. Without proper guidance, empathy is replaced by paralysis. Pierce [31] attributes this to gaps in life skills education, which rarely address neurological conditions, thereby perpetuating fear and exclusion.

These findings align with Goffman’s stigma theory and Erikson’s psychosocial model. Goffman [16] describes stigma as a discrediting label that transforms identity, while Erikson [17] emphasizes that middle childhood is when competence and self-esteem are built through social interaction. When stigma disrupts these processes, children internalize inferiority, compromising healthy identity formation.

As stigma accumulates, emotional and psychological harm intensifies. Children with epilepsy may experience anxiety, sadness, and withdrawal, often misinterpreted by peers as unreliability. Moser [32] identified this pattern as a key predictor of poor mental health, while Thompson [33] linked perceived stigma to reduced resilience and quality of life. Consequently, identity formation becomes characterized by secrecy and avoidance rather than confidence.

Epilepsy stigma in childhood thus operates as a self-sustaining ecosystem: misconceptions breed fear, fear drives exclusion, and exclusion deepens internalized shame. Yet, this cycle can be disrupted through structured life skills education that promotes empathy and accurate understanding. Hyland, Gallagher, and Connolly [34] demonstrate that such programs reduce fear-based stigma by empowering learners with both knowledge and emotional competence.

When children learn what epilepsy is and how to respond empathetically, seizures may become normalized rather than feared. Mugumbate [35] found that resilience improves when environments affirm rather than stigmatize. Inclusive education may reframe epilepsy as a learning opportunity about difference and care, benefiting all learners. Addressing both knowledge and emotion, life skills programs can transform stigma into understanding and strengthen collective resilience.

The sociocultural settings of Limpopo and Mpumalanga shape learners’ perceptions of epilepsy through communal beliefs that attribute it to witchcraft or ancestral punishment. Similar patterns across other low- and middle-income contexts reinforce stigma through cultural interpretation and limited health literacy [36,37]. While contextually grounded in South Africa, these findings are transferable to settings where cultural beliefs and poor education similarly shape children’s experiences.

Ultimately, epilepsy-related stigma reflects the social reproduction of fear but also the potential for transformation through education. The four themes, although distinct, represent facets of a single social process that intertwines perception, emotion, and identity. A whole-school approach that integrates knowledge, empathy, and emotional intelligence can foster a sense of belonging and identity growth beyond the constraints of stigma.

### Limitations

This study relied solely on the perspectives of primary school learners. While this child-centered focus provided valuable insight into peer-level stigma and social meaning-making, it did not capture the views of other key stakeholders, such as teachers, caregivers, or school health staff. The study’s cross-sectional design and small purposive sample may limit generalizability to other contexts. Future research should adopt multi-informant and longitudinal designs to provide a more holistic understanding of how epilepsy stigma operates within the broader educational and community environment.

## 5. Conclusions

The four themes derived from the focus groups illustrate that epilepsy stigma in primary schools is not merely a health issue but a developmental and educational challenge. Misconceptions foster fear; social exclusion breeds internalized stigma; emotional distress reveals gaps in health literacy; and isolation undermines mental health and identity formation. Research across contexts reinforces the idea that these patterns are systemic but modifiable. Life skills education, anchored in empathy, inclusion, and resilience, offers a practical path forward. By normalizing discussions about epilepsy and equipping children with emotional tools to respond compassionately, schools can play a transformative role in dismantling stigma and supporting healthy identity development.

Future research should focus on developing and evaluating school-based interventions that integrate epilepsy awareness and stigma reduction within life skills curricula. Longitudinal studies exploring the sustained effects of such programs on children’s mental health and identity formation could provide valuable insights. Comparative investigations between rural and urban school contexts may also inform adaptable, culturally grounded educational frameworks.

## Data Availability

The data supporting the findings of this study are not publicly available due to ethical restrictions protecting the privacy of child participants. De-identified data may be made available from the corresponding author on reasonable request and with permission from the University of Venda Research Ethics Committee.
